# Supplementary material for: Facile electron delivery from graphene template to ultrathin metal-organic layers for boosting CO2 photoreduction
Source: Nat Commun. 2021 Feb 5;12:813. doi: 10.1038/s41467-021-21084-9 (PMC7864970; doi:10.1038/s41467-021-21084-9)
Supplement: Supplementary file 1 — Supplementary Information [file 41467_2021_21084_MOESM1_ESM.pdf]

Supplementary Information

Jia-Wei Wang et al.

Supplementary Figures

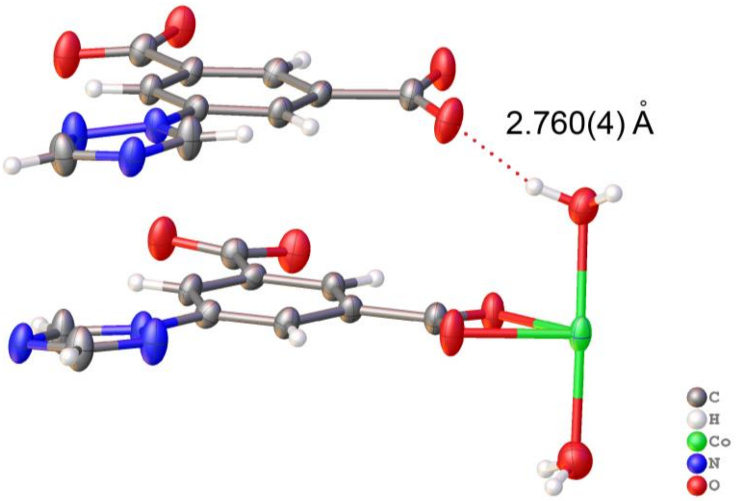

Supplementary Figure 1 | H-bonding mode. Schematic presentation of the H-bonding between two layers of Co-MOF.

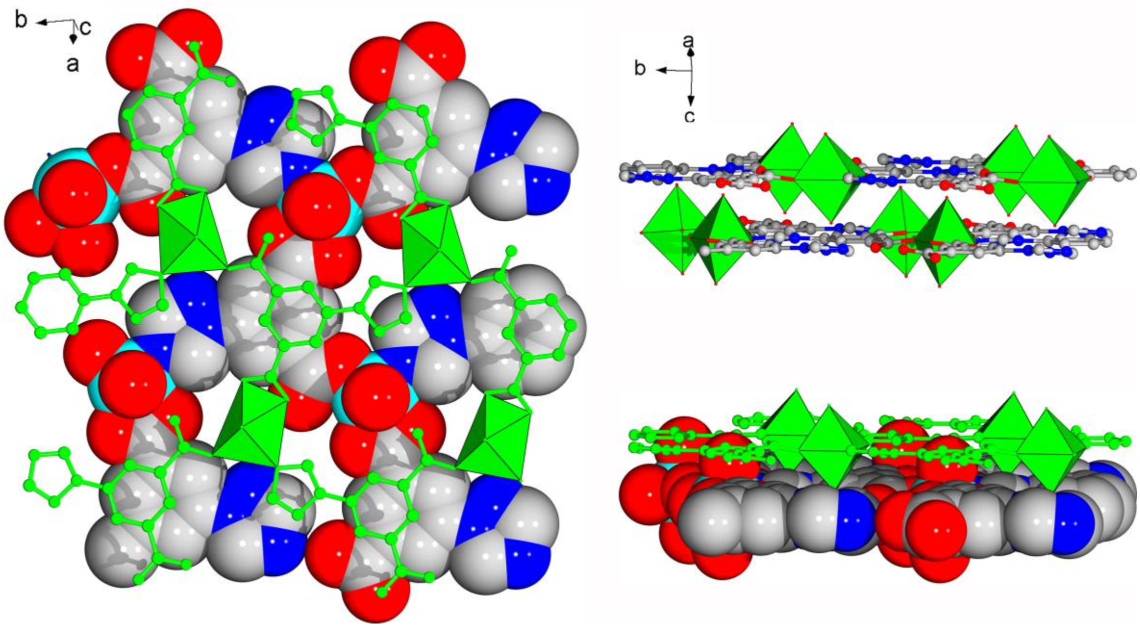

Supplementary Figure 2 | Crystallographic structure of Co-MOF. Presentation of crystallographic 3D structure of Co-MOF, stacked by the 2D metal-organic layers. The H atoms are omitted for clarity.

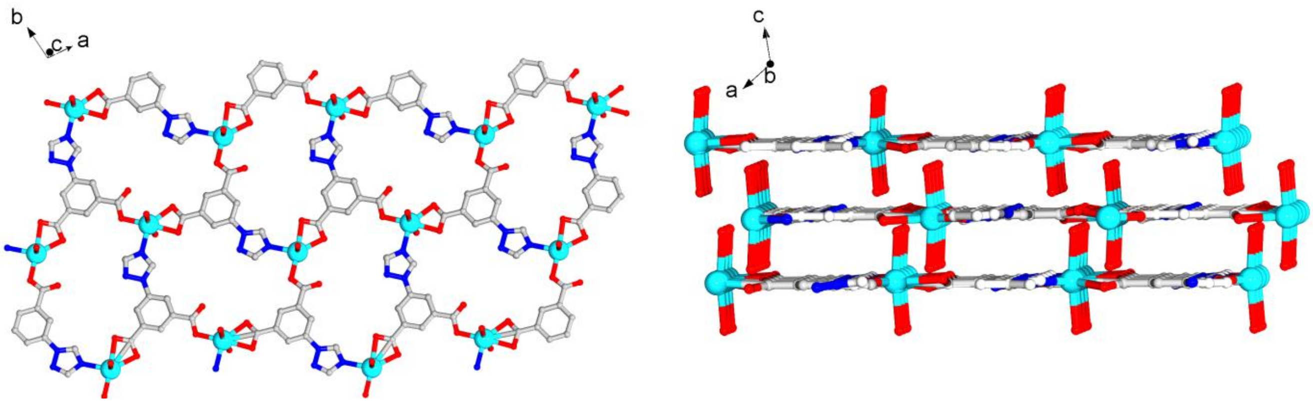

Supplementary Figure 3 | Crystallographic structure of Cd-MOF. Presentation of crystallographic 3D structure of Cd-MOF, stacked by the 2D metal-organic layers. The H atoms are omitted for clarity.

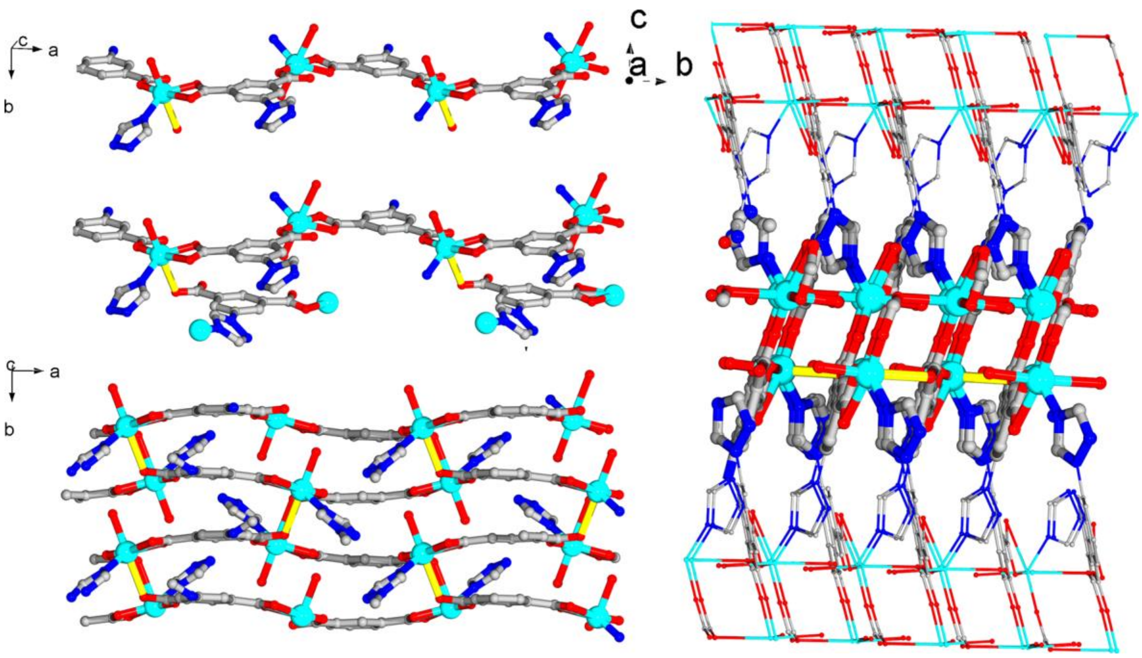

Supplementary Figure 4 | Crystallographic structure of Zn-MOF. Presentation of crystallographic 3D structure of Zn-MOF. The H atoms are omitted for clarity.

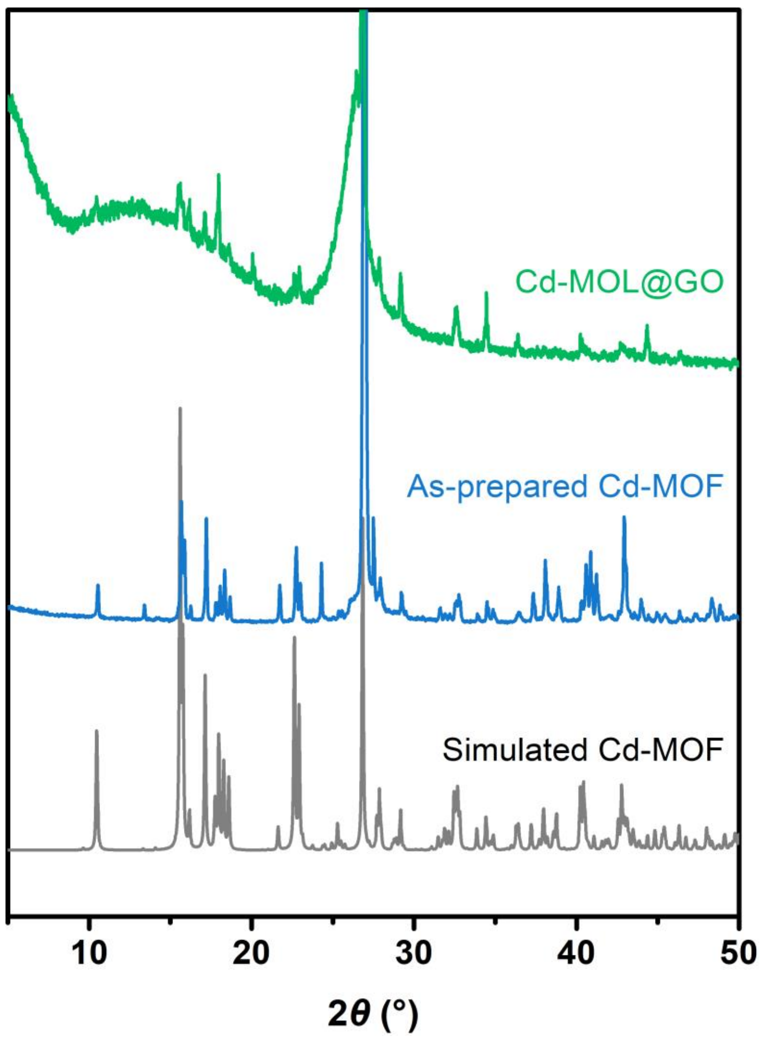

Supplementary Figure 5 | PXRD patterns for Cd-based samples. Simulated Cd-MOF (gray), as-prepared Cd-MOF (blue) and Cd-MOL@GO (green).

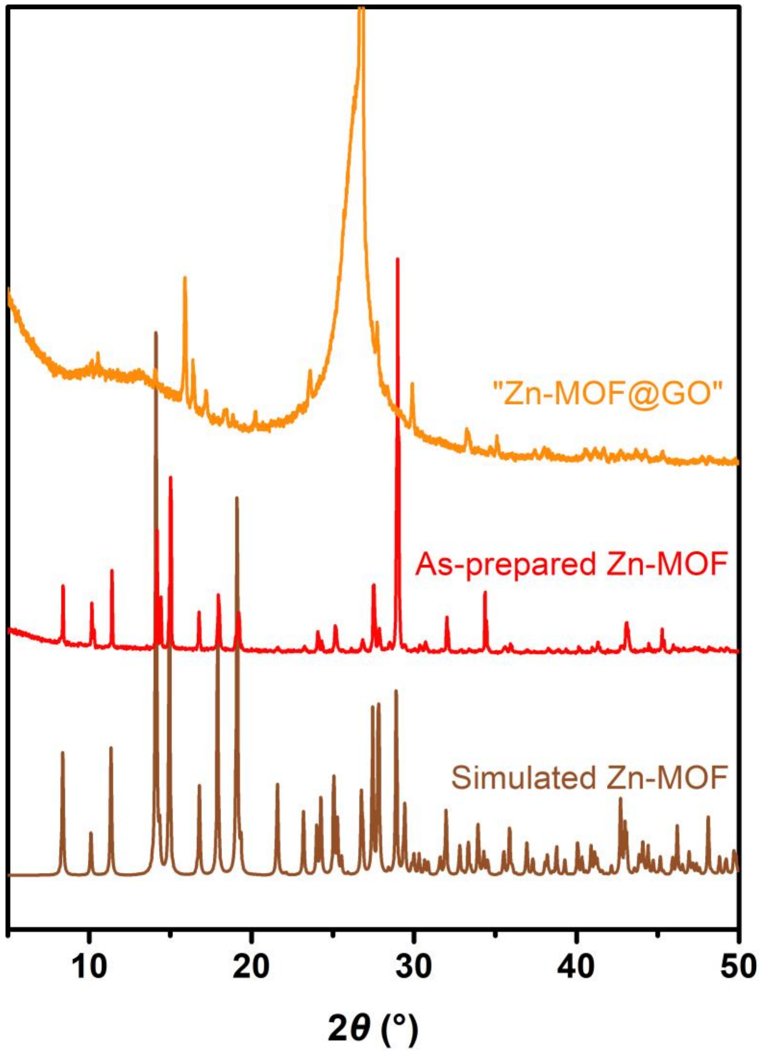

Supplementary Figure 6 | PXRD patterns for Zn-based samples. Simulated Zn-MOF (brown), as-prepared Zn-MOF (red), envisioned “Zn-MOF@GO” sample (orange).

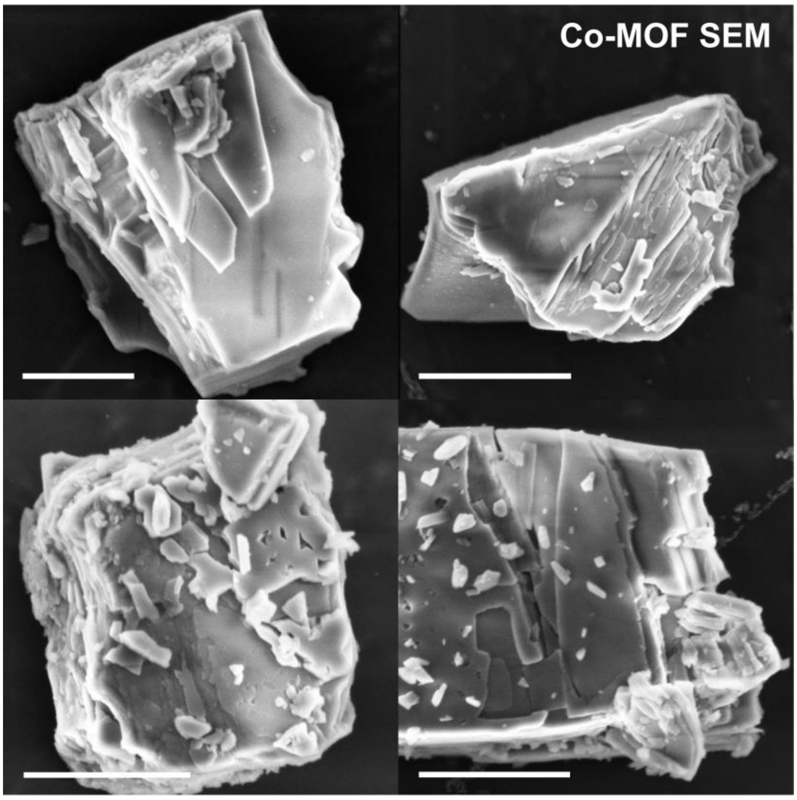

Supplementary Figure 7 | SEM. SEM images of bulky Co-MOF with the bar of 1 μm.

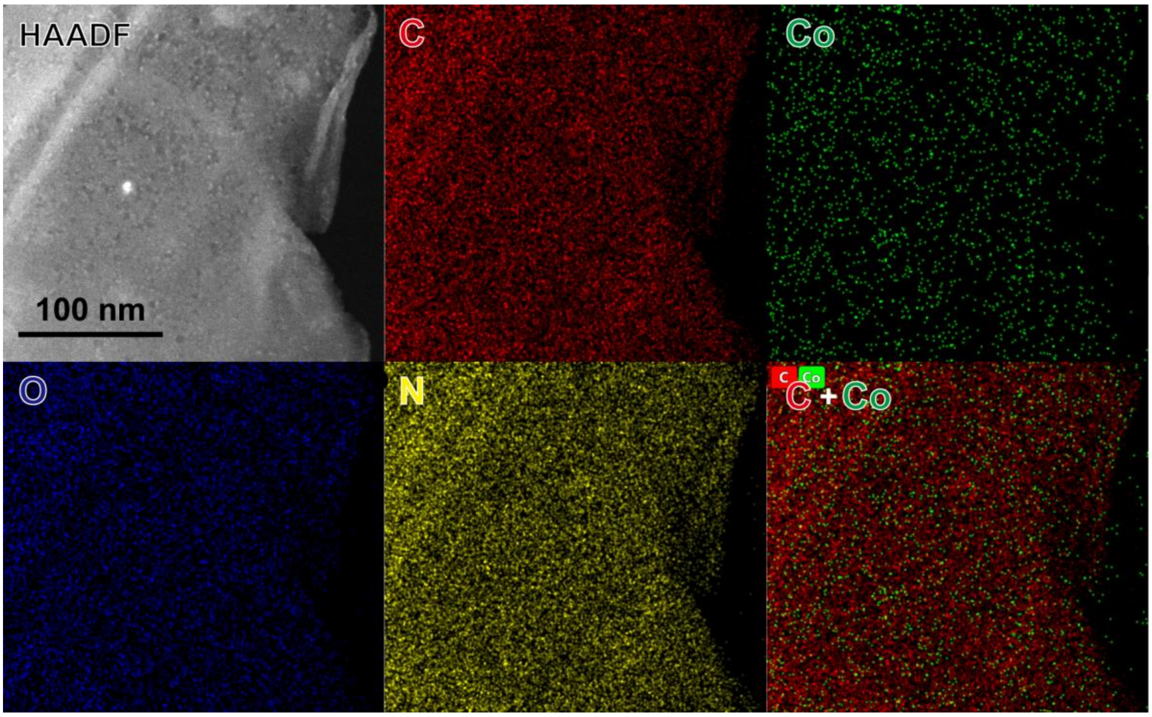

Supplementary Figure 8 | EDS mapping. EDS mapping of Co-MOL@GO, indicating the presence of Co and N elements on the GO.

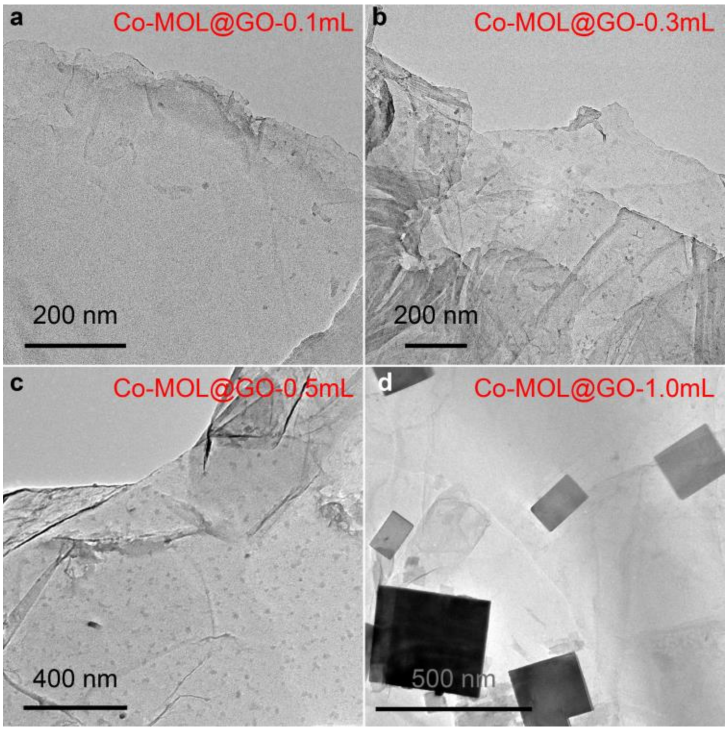

Supplementary Figure 9 | TEM for Co-MOL@GO with different loading amounts. TEM images for the Co-MOL@GO samples prepared with different loading amounts of Co^{2+}, including **a** 0.1, **b** 0.3, **c** 0.5 and **d** 1.0 mL aqueous solution of $CoCl_2$ $6H_2O$ (0.1 M).

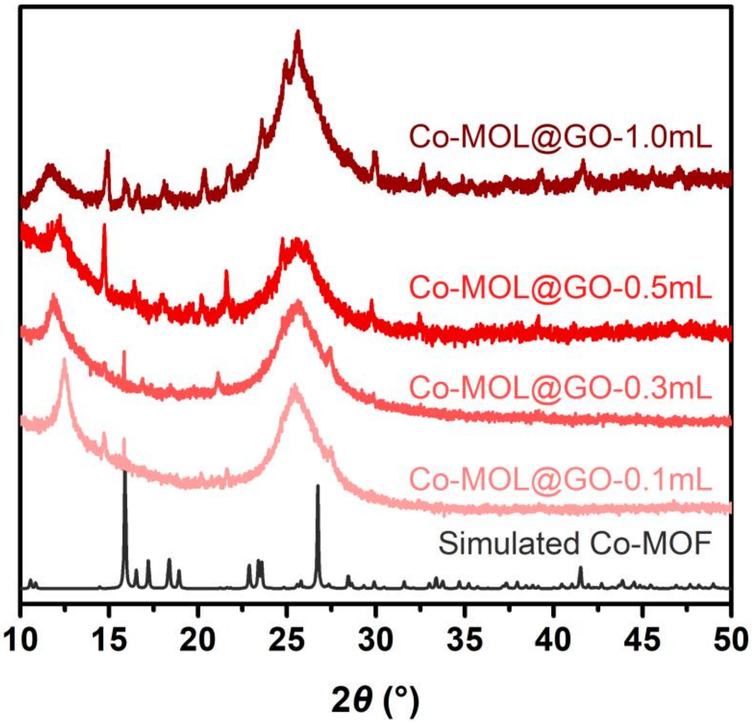

Supplementary Figure 10 | PXRD results for Co-MOL@GO with different loading amounts of Co^{2+}. PXRD patterns of the Co-MOL@GO samples prepared with 0.1, 0.3, 0.5 or 1.0 mL aqueous solution of $CoCl_2$ $6H_2O$ (0.1 M). The simulated PXRD pattern of Co-MOF is shown for comparison.

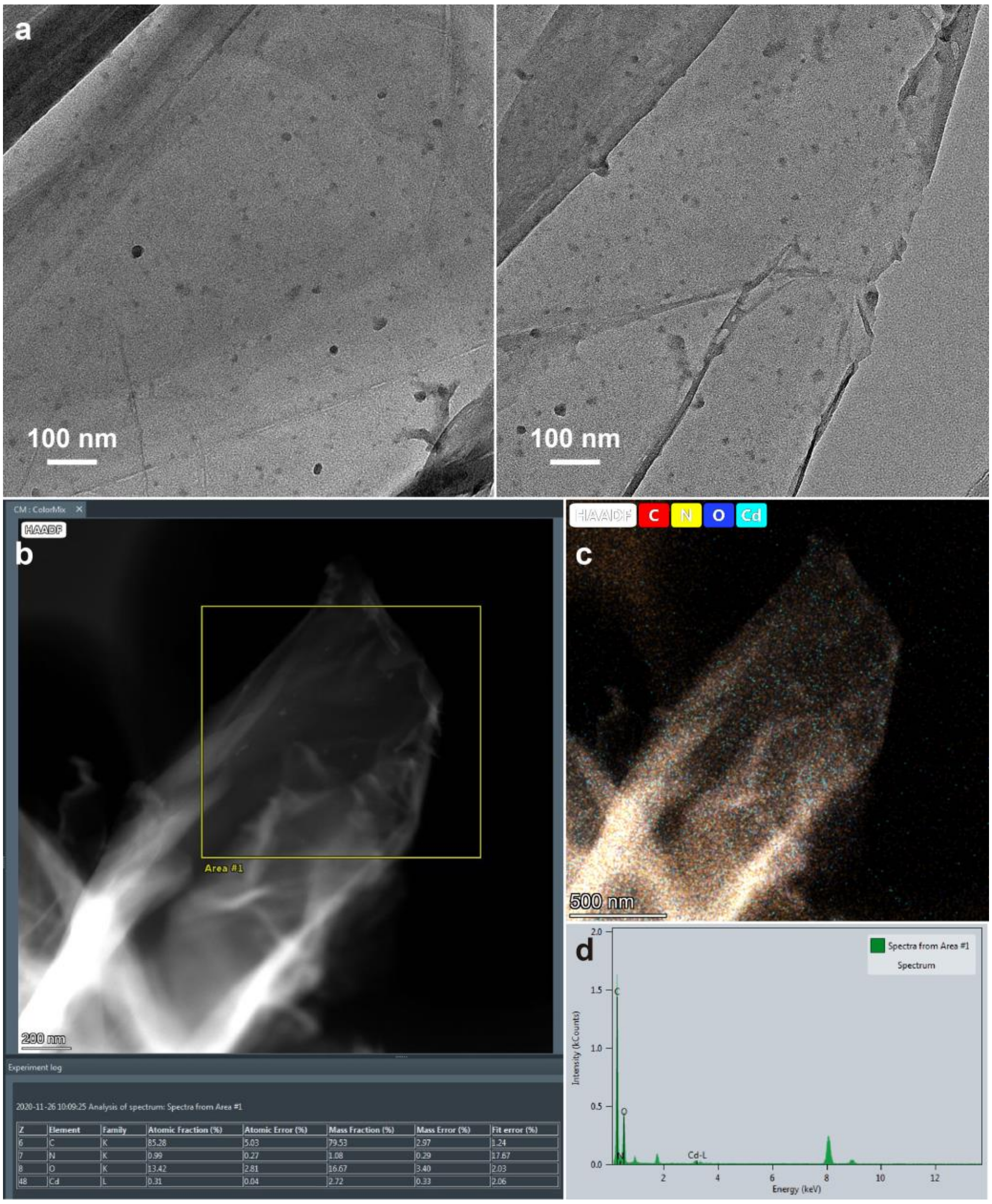

Supplementary Figure 11 | TEM and EDX characterizations on Cd-MOL@GO. a TEM images of Cd-MOL@GO, showing a range of MOL diameter of 20-30 nm. **b-d** EDX results of Cd-MOL@GO.

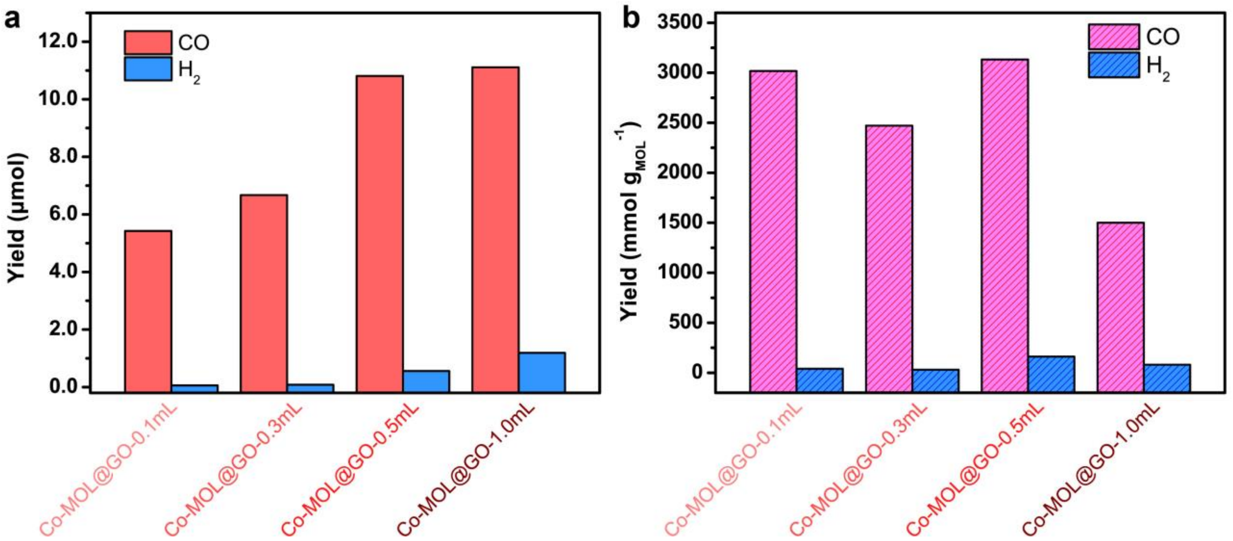

Supplementary Figure 12 | Photocatalysis with loading-varied Co-MOL@GO samples. Comparisons of CO and H_2 **a** yields in μmol or **b** yields in mmol g_{MOL}^{-1}, with 10 mg L^{-1} Co-MOL@GO samples prepared with 0.1, 0.3, 0.5 or 1.0 mL aqueous solution of $CoCl_2$ $6H_2O$ (0.1 M). Other conditions: 0.4 mM RuPS and 0.3 M TEOA in 5 mL CO_2-saturated CH_3CN/H_2O (v:v = 4:1) solution. With the comprehensive estimation of CO yield, Co-MOL@GO-0.5mL was chosen as the optimal catalyst.

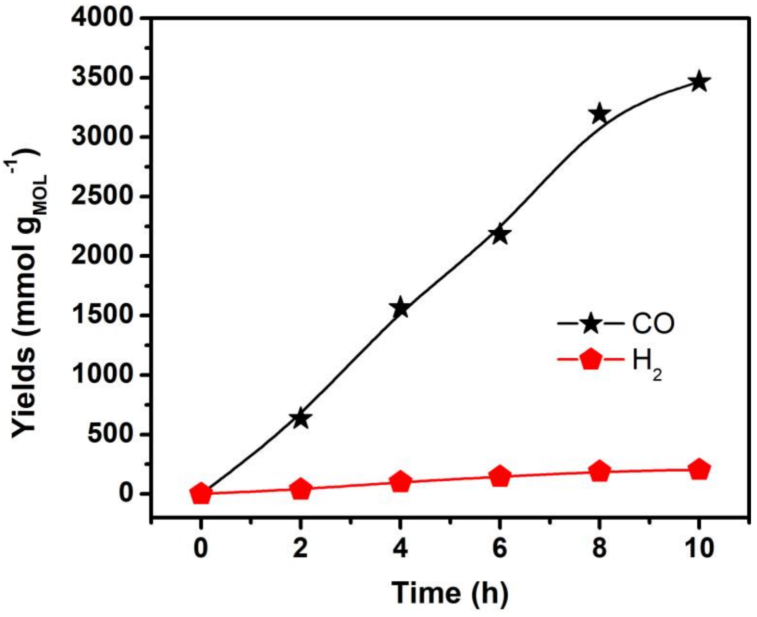

Supplementary Figure 13 | Photocatalysis. Time profiles of CO (black star) and H_2 (red pentagon) evolution catalyzed by 10 mg L^{-1} Co-MOL@GO in a five-times scaling-up reaction system.

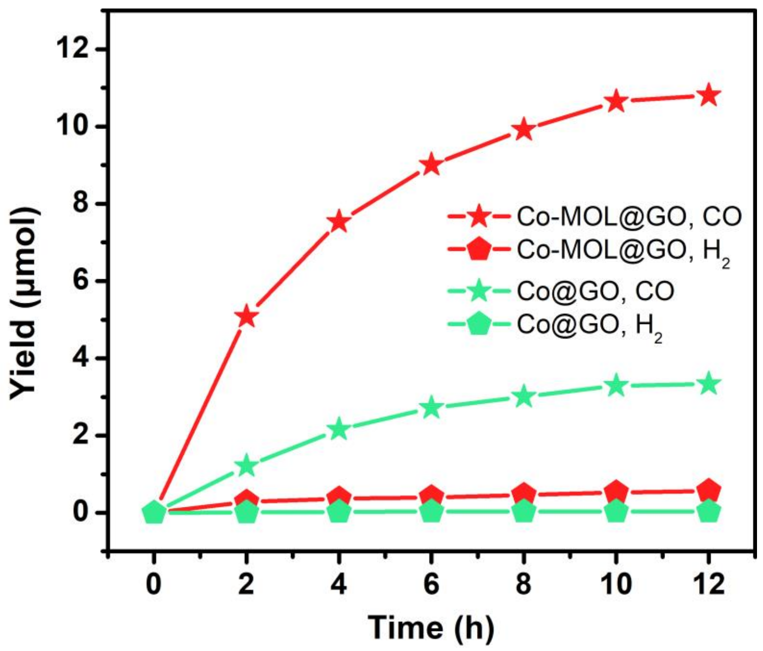

Supplementary Figure 14 | Photocatalysis. Time profiles of CO (star) and H_2 (pentagon) evolution catalyzed by 10 mg L^{-1} Co-MOL@GO (red) and Co@GO (green) under irradiation in the presence of 0.4 mM RuPS and 0.3 M TEOA in 5 mL CO_2-saturated CH_3CN/H_2O (v:v = 4:1) solution.

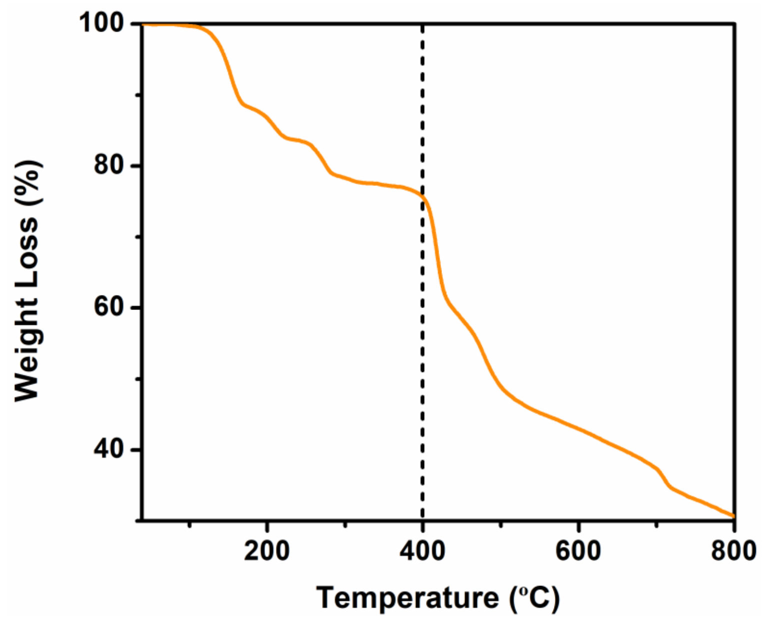

Supplementary Figure 15 | TGA. TGA curve of Co-MOF.

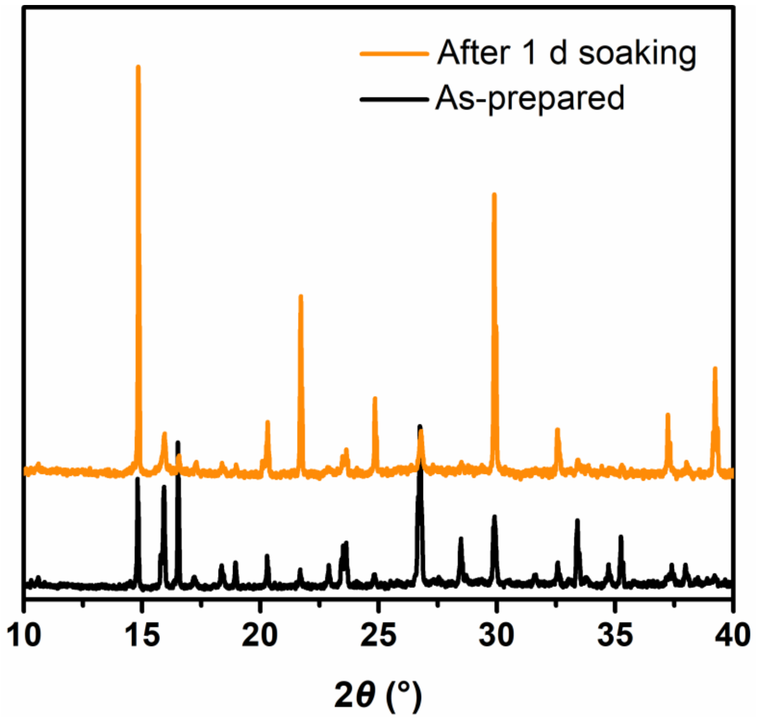

Supplementary Figure 16 | PXRD. PXRD patterns of as-prepared Co-MOF sample (orange) and that soaked in a CO_2-saturated CH_3CN/H_2O (v:v = 4:1) solution containing 0.3 M TEOA for 1 d (black).

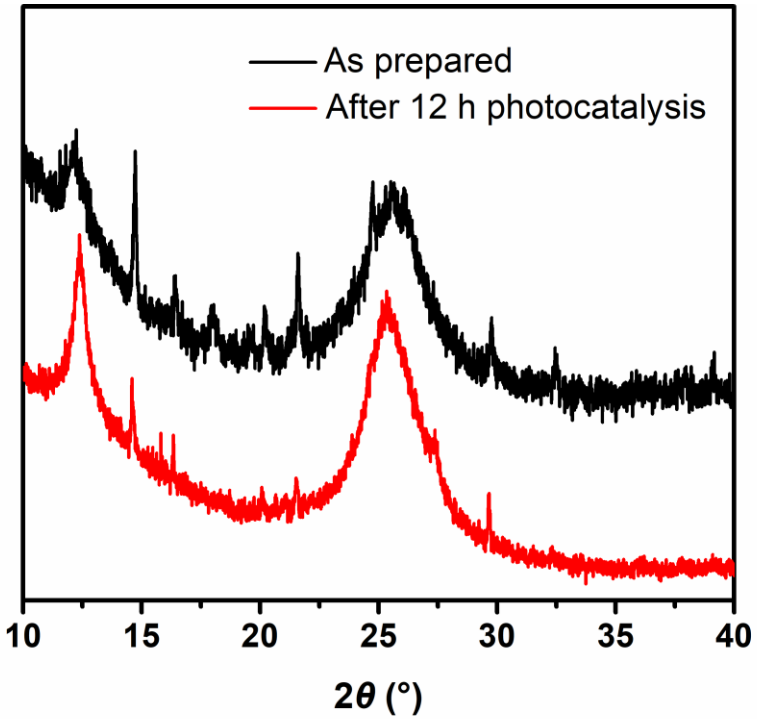

Supplementary Figure 17 | PXRD. PXRD patterns of as-prepared Co-MOL@GO (black) and the one experienced 12 h photo-reaction in a CO_2-saturated CH_3CN/H_2O (v:v = 4:1) solution containing 0.3 M TEOA (red).

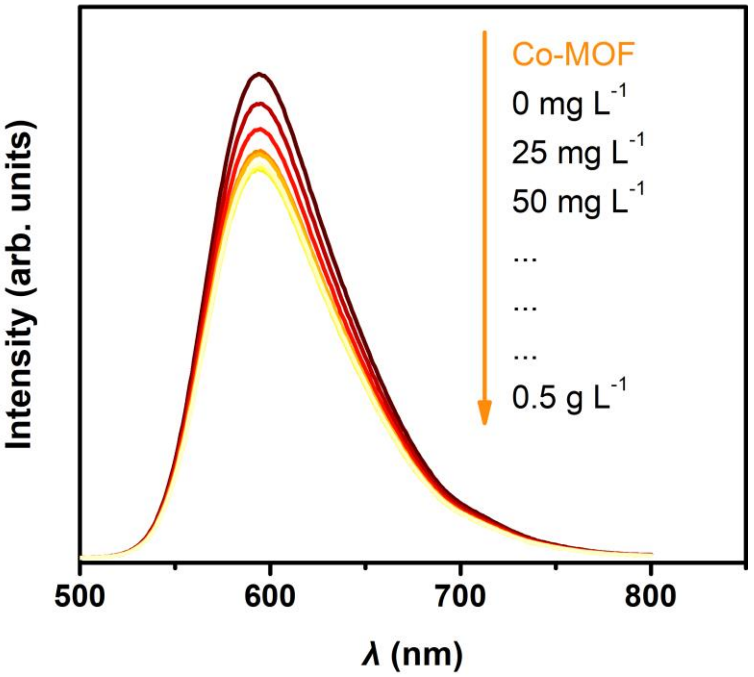

Supplementary Figure 18 | Quenching experiments. Fluorescence spectra of a CH_3CN/H_2O (*v*:*v* = 4:1) solution containing 0.4 mM RuPS in the presence of 0~0.5 g L^{-1} of Co-MOF.

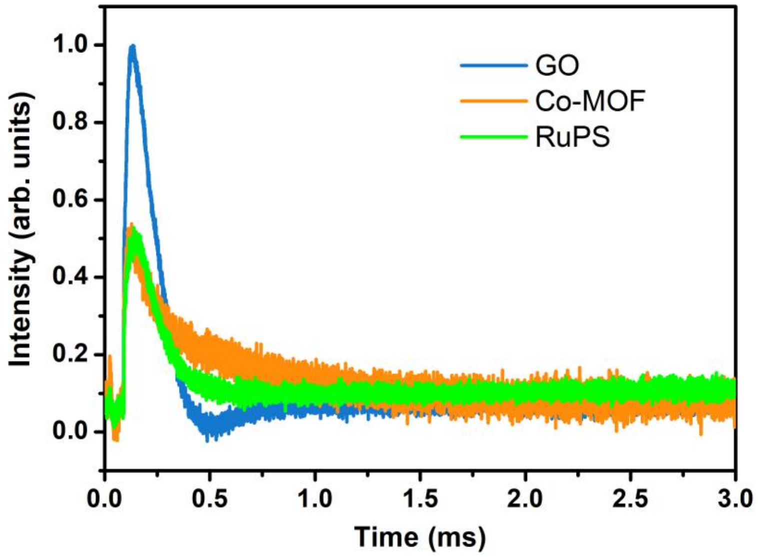

Supplementary Figure 19 | TPV. TPV curves of dry GO (blue), Co-MOF (orange) and RuPS (green) powders in air.

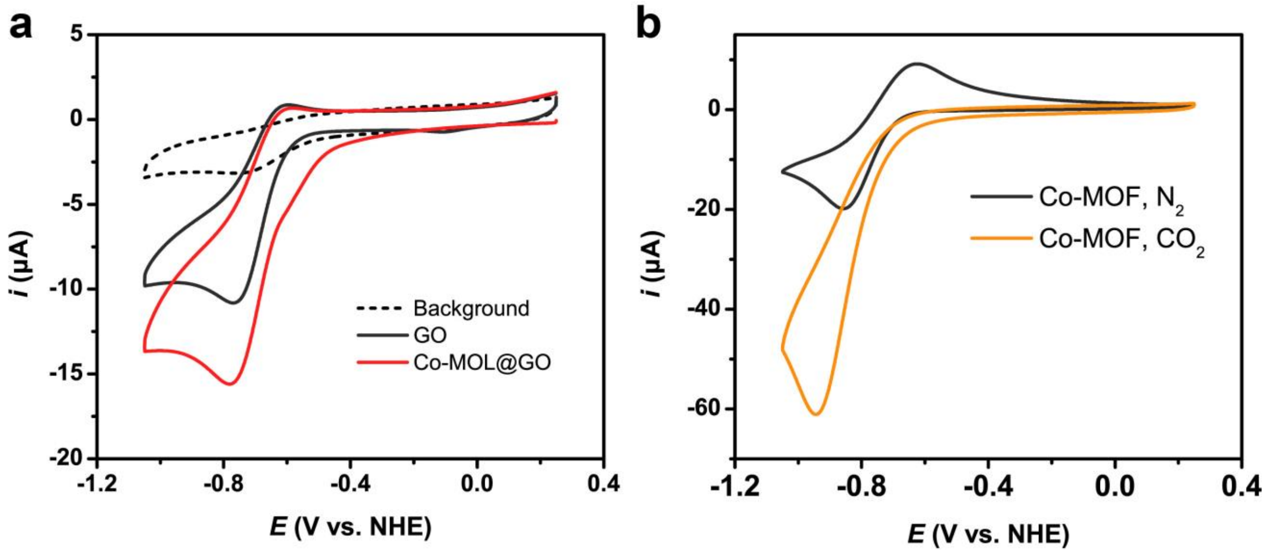

Supplementary Figure 20 | Cyclic voltammetry. a CVs of Co-MOL@GO (red) or GO (black solid line) under N_2, **b** CVs of Co-MOF under N_2 (black) or CO_2 (orange) on a glass carbon disk electrode (3 mm diameter) in CH_3CN/H_2O (v:v = 4:1) solution at 0.1 V s^{-1} scan rate. The CV obtained with bare glass carbon disk electrode is shown for comparison (black dashed line in Supplementary Figure 20a).

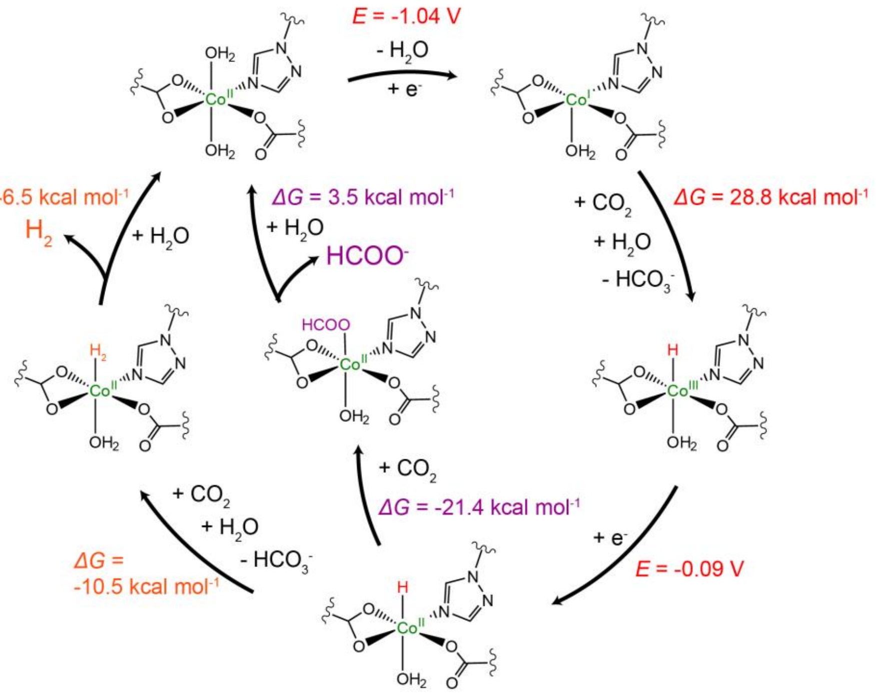

Supplementary Figure 21 | Calculated mechanism of Co-MOF. Calculated mechanism with the molecular unit of Co-MOF for catalytic proton reduction to H_2 and CO_2 reduction to formate, showing the calculated redox potentials and free energy changes.

Supplementary Tables

Supplementary Table 1 | Crystallographic data. Crystallographic data of Co-MOF, Cd-MOF and Zn-MOF.

Complex	Co-MOF	Cd-MOF	Zn-MOF
Formula	$CoC_{10}H_9N_3O_{6.5}$	$CdC_{10}H_{10}N_3O_{6.5}$	$ZnC_{10}H_8N_3O_{5.5}$
CCDC number	1965944	2047069	2047070
Formula weight	335.14	388.61	323.56
Crystal system	monoclinic	monoclinic	orthorhombic
Space group	*C*2/*c*	*C*2/*c*	*Pbcn*
Z	8	8	8
a / Å	19.6624(4)	19.9580(5)	15.5913(3)
b / Å	10.7223(2)	10.9485(3)	6.7209(2)
c / Å	13.4748(3)	13.4505(3)	21.1187(5)
β / °	124.166(2)	122.541(2)	90.00
V / Å^3	2350.55(8)	2477.66(11)	2212.98(9)
ρ_{calcd} / g m^{-3}	1.894	2.084	1.942
M / mm^{-1}	11.844	14.498	3.393
2θ range collected / °	4.94 / 79.34	9.64 / 134.14	8.38 / 158.88
Reflns collected/Indep.	8434 / 2484	7038 / 2208	7971 / 2374
R_{int}	0.0339	0.1073	0.0392
F(000)	1360.0	1528.0	1304.0
GOF on F^2	1.071	1.180	1.069
Final R indices [I > 2sigma(I)]	$^aR_1$ = 0.0427, $^bwR_2$ = 0.1244	R_1 = 0.0950, wR_2 = 0.2371	R_1 = 0.0448, wR_2 = 0.1340
R indices (all data)	R_1 = 0.0449, wR_2 = 0.1261	R_1 = 0.0970, wR_2 = 0.2390	R_1 = 0.0477, wR_2 = 0.1377

$^{[a]}R_1 = \sum||F_0| - |F_c||/\sum F_0$. $^{b}wR_2 = \{[\sum(F_0^2-F_c^2)/\sum w\,(F_0^2)^2]\}^{1/2}$

Supplementary Table 2 | Co amounts of Co-MOL@GO determined by ICP-MS and the photocatalytic performance.

Entry	V_{Co} (mL)[a]	Sample	Co contents (w%)	Co-MOL contents (w%)	CO/H_2 yields (μmol)[b]	CO/H_2 yields (mol g^{-1}_{MOL})[b]
1	0.1	Co-MOL@GO	0.51 ± 0.01	2.93	4.42/0.059	3017/40.3
2	0.3	Co-MOL@GO	0.94 ± 0.02	5.40	6.67/0.082	2471/30.2
3	0.5	Co-MOL@GO	1.20 ± 0.02	6.90	10.81/0.56	3133/162
4	1.0	Co-MOL@GO	2.41 ± 0.06	14.8	11.11/1.19	1501/80.4

[a]V_{Co} is the added volume of 1.0 M $CoCl_2$ solution.

[b]10 mg L^{-1} Co-MOL@GO was used for 10 h photocatalysis.

Supplementary Table 3 | Photocatalytic performances of MOF catalysts for CO_2 reduction to CO with Ru-based PSs.

Catalysts (mass)	Medium	PS (mass)	Products (μmol)	Maximum CO Yield (mmol g^{-1})	CO (%)	Ref
Co-MOL@GO<br>(0.05 mg)	CH_3CN (4.0 mL)<br>H_2O (1.0 mL)<br>TEOA (0.2 mL)	[Ru(phen)$_3$](PF$_6$)$_2$<br>(2.0 mg)	CO: 10.81<br>H_2: 0.56	216.1 (3133 based on MOL)	95	This Work
Co-MOF<br>(0.05 mg)			CO: 4.58<br>H_2: 1.0	91.5	82	
2D-Ni$_2$TCPE<br>(5 mg)	CH_3CN (48 mL)<br>H_2O (12 mL)<br>TEOA (6 mL)	[Ru(bpy)$_3$]Cl$_2$ 6H$_2$O<br>(37.5 mg)	CO: 100<br>H_2: 1.03	CO: 20<br>H_2: 0.2	97.2	1
MOF-Ni<br>(5 mg)	CH_3CN (28 mL)<br>H_2O (2 mL)<br>tri-isopropanolamine (TIPA; 2 mL)	[Ru(bpy)$_3$]Cl$_2$ 6H$_2$O<br>(7.5 mg)	CO: 22.3<br>H_2: 0.5	CO: 1.86<br>H_2: 0.04	97.7	2
CN-250-Fe$_2$Mn<br>(5 mg)	CH_3CN (45 mL)<br>H_2O (3 mL)<br>TIPA (10 mL)	[Ru(bpy)$_3$]Cl$_2$ 6H$_2$O<br>(60 mg)	CO: 430<br>CO: 94.4	86.04	82	3
MAF-X27l-OH<br>(1.77 mg)	CH_3CN (4.0 mL)<br>H_2O (1.0 mL)<br>TEOA (0.2 mL)	[Ru(bpy)$_3$]Cl$_2$ 6H$_2$O<br>(~1.5 mg)	CO: 45<br>H_2: 0.8	25.4	98	4
Zr-DMBD-Co<br>(0.1 mg)	CH_3CN (4.0 mL)<br>H_2O (1.0 mL)<br>TEOA (0.2 mL)	[Ru(phen)$_3$](PF$_6$)$_2$<br>(2.0 mg)	CO: 3.33<br>H_2: 0.041	33.3	99	5
Co-ZIF-9<br>(0.2 mg)<br>(3 mg)	CH_3CN (4.0 mL)<br>H_2O (1.0 mL)<br>TEOA (1.0 mL)	[Ru(bpy)$_3$]Cl$_2$ 6H$_2$O<br>(~7.5 mg)	CO: 41.8<br>H_2: 30.29<br>CO: 51.6<br>H_2: 47.63	209<br>17.2	58<br>52	6
ZIF-67_3	CH_3CN (4.0 mL)	[Ru(bpy)$_3$]Cl$_2$ 6H$_2$	CO: 16	1.6	63	7

(10 mg)	H_2O (1.0 mL)<br>TEOA (1.0 mL)	O<br>(8 mg)	H_2: 9			
Ni(TPA/TEG)<br>(3 mg)	CH_3CN (8.0 mL)<br>H_2O (2.0 mL)<br>TEOA (2.0 mL)	$[Ru(bpy)_3]Cl_2$ 6H_2<br>O<br>(1.5 g)	CO: 140	47	99	8
$Ni_3(HITP)_2$<br>(2 mg)<br>At 4 C°, 80 kPa CO_2	CH_3CN (8.0 mL)<br>H_2O (2.0 mL)<br>TEOA (4.0 mL)	$[Ru(bpy)_3]Cl_2$ 6H_2<br>O<br>(80 mg)	CO: 207<br>H_2: 7.49	103.5	97	9
Ni MOLs<br>(1 mg)	CH_3CN (3.0 mL)<br>H_2O (2.0 mL)<br>TEOA (1.0 mL)	$[Ru(bpy)_3]Cl_2$ 6H_2<br>O<br>(7.5 mg)	CO: 25<br>H_2: 0.56	25	98	10
Co ZIF-8<br>(1.0 mg)<br>(0.1 mg)	CH_3CN (3.0 mL)<br>H_2O (2.0 mL)<br>TEOA (1.0 mL)	$[Ru(bpy)_3]Cl_2$ 6H_2<br>O<br>(8 mg)	CO: 26.6<br>H_2: 14.8<br>CO: 37.4<br>H_2: 13	26.6<br>374	67<br>74	11
Zn ZIF-8<br>(1.0 mg)	CH_3CN (3.0 mL)<br>H_2O (2.0 mL)<br>TEOA (1.0 mL)	$[Ru(bpy)_3]Cl_2$ 6H_2<br>O<br>(8 mg)	CO: 1.8<br>H_2: 2.0	18	47	11
Cu HKUST-1<br>(1 mg)	CH_3CN (3.0 mL)<br>H_2O (2.0 mL)<br>TEOA (1.0 mL)	$[Ru(bpy)_3]Cl_2$ 6H_2<br>O<br>(8 mg)	CO: 1.5<br>H_2: 1.91	1.5	44	11
Zr-UIO-66-NH_2<br>(1 mg)	CH_3CN (3.0 mL)<br>H_2O (2.0 mL)<br>TEOA (1.0 mL)	$[Ru(bpy)_3]Cl_2$ 6H_2<br>O<br>(8 mg)	CO: 0.9<br>H_2: 1.24	0.9	43	11
Fe-MIL-101-NH_2<br>(1 mg)	CH_3CN (3.0 mL)<br>H_2O (2.0 mL)<br>TEOA (1.0 mL)	$[Ru(bpy)_3]Cl_2$ 6H_2<br>O<br>(8 mg)	CO: 4.7<br>H_2: 2.1	4.7	69	11

Supplementary Table 4 | Relative free energy in kcal mol^{-1} for intermediates at different spin states.[a]

Entry	Intermediates	singlet	doublet	triplet	quartet
1	Co^{II}	N.A.	23.32	N.A.	0
2	Co^{I}	22.62	N.A.	0	N.A.
3	Co^{II}-CO_2	N.A.	0.08	N.A.	0
4	Co^{II}-COOH	N.A.	0	N.A.	0.05
5	Co^{II}-CO	N.A.	11.28	N.A.	0
6	Co^{III}-H	7.85	N.A.	0	N.A.
7	Co^{II}-H	N.A.	1.13	N.A.	0
8	Co^{II}-H_2	N.A.	20.97	N.A.	0
9	Co^{II}-HCOO	N.A.	18.30	N.A.	0

[a] For each lowest energy spin state, the free energy is set as reference point, 0 kcal mol^{-1}.

Supplementary Table 5 | Calculated $Co^{II/I}$ reduction potentials by different functional with def2SVP basis set.

Entry	Method/Functional	$Co^{II/I}$ reduction potential (V vs. NHE)
1	Measured value	-0.94
2	B3P86	-1.04
3	B3P86-D3	-1.16
4	M06-D3-	-1.60
5	M06-L-D3	-1.42
7	M06	-1.57
8	M06-L	-1.41
9	B3LYP	-1.56
10	B3LYP-D3	-1.68

Supplementary References

1. Zheng, H. L., *et al.* Photochemical in situ exfoliation of metal-organic frameworks for enhanced visible-light-driven CO_2 reduction. *Angew. Chem. Int. Ed.* DOI: 10.1002/anie.202012019 (2020), .
2. Wang, X.-K., *et al.* Monometallic catalytic models hosted in stable metal–organic frameworks for tunable CO_2 photoreduction. *ACS Catal.* 1726-1732 (2019).
3. Dong, H., *et al.* Regulation of metal ions in smart metal-cluster nodes of metal-organic frameworks with open metal sites for improved photocatalytic CO_2 reduction reaction. *Appl. Catal. B-Environ* DOI: 10.1016/j.apcatb.2020.119173 (2020).
4. Wang, Y., Huang, N. Y., Shen, J. Q., Liao, P. Q., Chen, X. M. & Zhang, J. P. Hydroxide ligands cooperate with catalytic centers in metal-organic frameworks for efficient photocatalytic CO_2 reduction. *J. Am. Chem. Soc.* **140**, 38-41 (2018).
5. Liu, D. C., *et al.* Anchoring Co(II) ions into a thiol-laced metal-organic framework for efficient visible-light-driven conversion of CO_2 into CO. *ChemSusChem* **12**, 2166-2170 (2019).
6. Wang, S., Yao, W., Lin, J., Ding, Z. & Wang, X. Cobalt imidazolate metal-organic frameworks photosplit CO_2 under mild reaction conditions. *Angew. Chem. Int. Ed.* **53**, 1034-1038 (2014).
7. Wang, M., *et al.* Metal–organic frameworks (ZIF-67) as efficient cocatalysts for photocatalytic reduction of CO_2: The role of the morphology effect. *J. Mater. Chem. A* **6**, 4768-4775 (2018).
8. Niu, K., *et al.* A spongy nickel-organic CO_2 reduction photocatalyst for nearly 100% selective CO production. *Sci. Adv.* **3**, e1700921 (2017).
9. Zhu, W., *et al.* Selective reduction of CO_2 by conductive MOF nanosheets as an efficient co-catalyst under visible light illumination. *Appl. Catal. B-Environ* **238**, 339-345 (2018).
10. Han, B., *et al.* Nickel metal-organic framework monolayers for photoreduction of diluted CO_2: Metal-node-dependent activity and selectivity. *Angew. Chem. Int. Ed.* **57**, 16811-16815 (2018).
11. Qin, J., Wang, S. & Wang, X. Visible-light reduction CO_2 with dodecahedral zeolitic imidazolate framework ZIF-67 as an efficient co-catalyst. *Appl. Catal. B-Environ* **209**, 476-482 (2017).
